# Moss Cover Redirects Soil Organic Carbon from Active Turnover to Mineral-Associated Stabilization in Subalpine Forests

**DOI:** 10.3390/plants15132098

**Published:** 2026-07-06

**Authors:** Jiahui Huang, Xiaoyu Zhang, Yu Tian, Guo Luo, Dajun Xie, Jinxiao Li, Baoli Duan, Shuming Peng

**Affiliations:** 1 College of Environment and Ecology, Chengdu University of Technology, Chengdu 610059, China; m13073171217@163.com (J.H.); 18161076685@163.com (X.Z.); 14726838754@163.com (Y.T.); 18181777050@163.com (G.L.); 2 Sichuan Academy of Forestry, Chengdu 610081, China; 13808183024@163.com (D.X.); lijinxiaoforestry@126.com (J.L.); 3 Institute of Mountain Hazards and Environment, Chinese Academy of Sciences, Chengdu 610213, China; duanbl@imde.ac.cn

**Keywords:** moss cover, subalpine forests, microbial necromass carbon, mineral-associated organic carbon

## Abstract

Understory mosses modify near-surface soil conditions, but how elevation regulates their influence on active and mineral-associated soil organic carbon (SOC) remains unclear. We compared independently selected moss-covered and non-moss-covered soils across a 3200–3500 m elevational gradient and integrated soil physicochemical measurements, microbial biomass (MB), dissolved organic matter (DOM), microbial necromass carbon (MNC), particulate organic carbon (POC), mineral-associated organic carbon (MAOC), metagenomic profiling, and piecewise structural equation modeling. Moss-covered soils consistently contained higher SOC and MAOC, but lower DOM, MB, and generally lower POC, than non-moss-covered soils. MNC showed an elevation-dependent reversal, with higher values under moss cover at 3200 m but lower values under moss cover at 3300–3500 m. Elevation was not a significant uniform driver of MB, DOM, MNC, POC, or MAOC; instead, its influence was mainly reflected in interactions with surface cover and in elevation-related changes in moss-layer structure, diversity, and hydrothermal conditions. Core carbon-fixation and degradation functions remained broadly stable, whereas specific functional modules shifted within moss-covered soils: acetate and acetyl-CoA metabolism genes (*ackA* and *abfD*) were relatively abundant at 3300–3400 m, while the polysaccharide-reprocessing gene *SGA1* and oxidative-transformation gene *katG* increased toward higher elevations, and *pmoC*/*amoC* rebounded at 3500 m. Structural equation models linked the microbial functional gene system more strongly to POC, whereas MNC was positively associated with MAOC, and the direct POC-to-MAOC pathway was not significant. These findings indicate that moss cover is associated with contrasting SOC allocation patterns and stronger microbial necromass–MAOC coupling, while elevation modulates these relationships indirectly through changes in moss communities, soil microenvironment, and microbial functional potential.

## 1. Introduction

Soil organic carbon (SOC) represents the largest carbon pool in terrestrial ecosystems and an important component of the global carbon cycle and climate feedbacks [1,2,3,4]. Forests are major terrestrial carbon sinks, with nearly half of forest ecosystem carbon stored in soils [5,6]. Therefore, identifying the biological and physicochemical mechanisms governing SOC persistence and stabilization is essential for predicting the long-term carbon sink capacity of forests under environmental change.

High-elevation montane coniferous forests, particularly subalpine spruce forests, represent important regional carbon reservoirs because low temperatures and short growing seasons generally suppress organic matter decomposition and promote SOC accumulation [7,8]. However, these ecosystems are highly sensitive to environmental heterogeneity along elevational gradients, including variations in temperature, moisture, radiation, and nutrient availability. Moreover, the understory microclimate beneath forest canopies can be substantially decoupled from regional macroclimatic conditions [9,10,11,12,13]. Such fine-scale environmental heterogeneity can influence microbial activity, substrate turnover, and SOC stabilization, highlighting the importance of ground-layer vegetation in shaping soil carbon dynamics.

Mosses are ubiquitous and functionally important components of subalpine forest floors. Their high water-holding capacity and low thermal conductivity enable moss layers to buffer fluctuations in soil temperature, retain moisture, and reduce near-surface evaporative water loss [14,15,16]. Moss cover can also intercept litter and atmospheric deposition, influence litter decomposition and nutrient release, and modify the transport of dissolved organic matter into mineral soil. By regulating substrate availability, oxygen diffusion, and hydrothermal conditions, moss layers create distinctive microbial habitats that may alter microbial community composition, biomass, and metabolic activity.

Evidence from cold and montane ecosystems further indicates that mosses are not merely passive components of the forest floor but function as ecosystem engineers. In boreal and Arctic ecosystems, mosses strongly regulate energy exchange, soil thermal regimes, hydrological processes, nutrient availability, and carbon cycling [17]. A global synthesis showed that soil mosses are associated with greater soil carbon sequestration, larger nutrient pools, and altered organic matter decomposition rates compared with adjacent bare soils [18]. At a broader biogeochemical scale, cryptogamic covers, including bryophytes, make substantial contributions to terrestrial carbon uptake and biological nitrogen fixation [18]. Recent studies published in Plants have also demonstrated that bryophyte hydration is closely related to elevation-dependent changes in temperature, precipitation, and relative humidity [19], and that moss presence can modify forest-floor CO_2_ fluxes and the isotopic characteristics of respired carbon [20]. Together with evidence that mosses influence litter decomposition, microbial community composition, and soil carbon and nitrogen accumulation in cold ecosystems [16,18,21,22], these findings suggest that moss cover may regulate SOC not only through direct carbon inputs but also by modifying microbial habitats and substrate-processing pathways. Nevertheless, how moss cover is associated with microbial-derived carbon formation, SOC stabilization pathways, and carbon pool redistribution along subalpine elevational gradients remains insufficiently understood.

Contemporary SOC stabilization theory increasingly emphasizes microbial transformation and physicochemical protection rather than the intrinsic chemical recalcitrance of plant-derived substrates [23,24]. The Microbial Carbon Pump framework proposes that microorganisms assimilate and transform plant-derived carbon into microbial biomass and, following microbial turnover, into microbial necromass that can subsequently be retained through mineral adsorption or aggregate occlusion [25,26,27]. Microbial anabolic activity, carbon use efficiency, and necromass accumulation therefore provide important links between relatively labile organic inputs and the formation of persistent SOC [28,29,30].

These stabilization processes can be evaluated by separating SOC into particulate organic carbon (POC) and mineral-associated organic carbon (MAOC). POC is composed primarily of partially decomposed plant residues and generally has a relatively rapid turnover rate, whereas MAOC contains a greater proportion of microbially processed compounds protected through organo-mineral associations and contributes disproportionately to long-term SOC persistence [28,31,32,33]. Microbial necromass carbon (MNC) is increasingly recognized as an important precursor and component of MAOC [34,35,36]. Meanwhile, soil metagenomics provides insights into the functional potential of microbial communities involved in carbon fixation, decomposition, assimilation, and transformation [37,38,39,40]. Integrating metagenomic functional profiles with MNC measurements and SOC fractionation can therefore help clarify how microbial carbon-cycling potential is associated with the formation and stabilization of distinct soil carbon pools [41].

In this study, we compared independently selected moss-covered and non-moss-covered soils within four elevational bands along a 3200–3500 m gradient in a subalpine dark coniferous forest. We integrated moss-layer traits, soil hydrothermal and nutrient conditions, SOC fractionation, microbial necromass carbon, metagenomic carbon-cycling profiles, and piecewise structural equation modeling within a unified analytical framework. The distinctive contribution of this study is that it evaluates the microbial functional gene system as a key link between elevation-related variation in moss communities and belowground carbon processing, while separately examining the association of microbial necromass with MAOC. This framework allowed us to distinguish microbial carbon-cycling potential from carbon stabilization outcomes and to test whether MAOC was more closely associated with microbial necromass than with direct POC-to-MAOC transformation. We hypothesized that: (1) moss cover would modify near-surface hydrothermal conditions and nutrient availability along the elevational gradient; (2) moss cover would alter SOC fraction allocation, with generally lower POC and higher MAOC under moss-covered conditions; and (3) microbial functional potential and microbial necromass would show distinct associations with POC and MAOC, respectively.

## 2. Results

### 2.1. Variations in Moss-Layer Characteristics and α- and β-Diversity

Moss-layer characteristics, community α-diversity, and compositional similarity varied markedly along the altitudinal gradient (Figure S1; Tables S1 and S2). Moss biomass increased with elevation and was significantly higher at 3400 and 3500 m than at 3200 and 3300 m (*p* < 0.05; Figure S1a). In contrast, moss-layer thickness generally decreased with increasing elevation, although the pattern was not strictly monotonic. Specifically, moss-layer thickness was highest at 3200 m, decreased at 3300 m, slightly increased at 3400 m, and reached the lowest value at 3500 m (Figure S1b). Moss biomass and moss-layer thickness showed different elevational patterns. Moss community α-diversity also showed clear altitudinal responses. Chao1 richness and Shannon diversity were highest at 3200 m and generally decreased toward higher elevations, with particularly low values observed at 3500 m, although the trends were not strictly monotonic among intermediate elevations (Figure S1d,f). Dahl’s similarity index, as a descriptive β-diversity-related measure of compositional similarity, was highest at 3200 m and generally decreased toward higher elevations, indicating reduced compositional similarity of moss communities along the elevational gradient (Figure S1c). In contrast, the Simpson index increased toward the highest elevation and was significantly higher at 3500 m than at the low- and mid-elevation sites (*p* < 0.001; Figure S1e).

Pairwise β-diversity further indicated compositional differences in moss communities among elevations (Tables S1 and S2). The Cody β-diversity index (βC) ranged from 7.11 to 10.50, with the highest species turnover observed between 3200 and 3400 m and the lowest between 3400 and 3500 m (Table S1). The Wilson-Shmida β-diversity index (βT) ranged from 0.64 to 1.03, with relatively high turnover between 3200 and 3500 m, whereas the lowest value occurred between 3200 and 3300 m (Table S2). These results suggest that moss community composition differed among elevations, and the magnitude of species turnover varied among elevation pairs.

Overall, moss biomass increased with elevation, whereas moss-layer thickness and most α-diversity indices decreased. In addition, Dahl’s similarity index and pairwise β-diversity analyses indicated evident compositional differentiation and species turnover among elevations, further supporting altitudinal variation in moss community structure.

### 2.2. Changes in Soil Physicochemical Properties

Soil chemical properties showed distinct elevation- and cover-dependent patterns (Table S3). SOC varied only slightly with elevation in non-moss-covered soils, whereas in moss-covered soils it generally decreased with increasing elevation, reaching the highest level at 3200 m and the lowest at 3500 m. At each elevation, SOC was significantly higher in moss-covered soils than in non-moss-covered soils, with the most pronounced difference observed at 3200 m. The elevational pattern of total nitrogen (TN) differed between cover treatments: in non-moss-covered soils, TN was higher at lower elevations and lower at higher elevations, whereas in moss-covered soils it reached a relatively high level at 3300 m and decreased markedly at 3500 m. Moss cover significantly reduced TN at 3200 and 3500 m. Total phosphorus also showed clear elevational variation. In non-moss-covered soils, TP was relatively high at 3400 m, whereas in moss-covered soils it was higher at 3300 m and lowest at 3500 m. Compared with non-moss-covered soils, moss cover significantly reduced TP at 3400 and 3500 m. Nitrate-N and Available Phosphorus generally remained at relatively high levels in non-moss-covered soils but were markedly reduced under moss cover; at each elevation, both nitrate-N and AP were significantly lower in moss-covered soils than in non-moss-covered soils. DOM showed little variation along the elevational gradient in non-moss-covered soils and remained consistently high, whereas in moss-covered soils it was generally much lower and increased with elevation, reaching the highest level at 3500 m.

Soil physical properties showed distinct elevational patterns, and moss-covered soils generally had lower mean soil temperature and pH, but higher ΔT and soil water content, than non-moss-covered soils (Table S4). Mean soil temperature generally increased with elevation in non-moss-covered soils and reached its highest level at 3500 m. In contrast, in moss-covered soils, mean temperature first decreased with increasing elevation, reached its lowest level at 3400 m, and then increased slightly at 3500 m. Within each elevation, mean soil temperature was significantly lower in moss-covered soils than in non-moss-covered soils (*p* < 0.05). ΔT generally decreased with increasing elevation in both cover treatments. In non-moss-covered soils, ΔT continuously decreased from 3200 to 3500 m, while in moss-covered soils it also declined from the highest level at 3200 m to the lowest level at 3500 m. Within each elevation, ΔT was significantly higher in moss-covered soils than in non-moss-covered soils (*p* < 0.05). Soil pH changed only slightly along the elevational gradient in non-moss-covered soils and remained relatively stable. However, in moss-covered soils, pH showed a fluctuating pattern with increasing elevation, increasing from 3200 to 3300 m, decreasing at 3400 m, and then increasing again at 3500 m. Within-elevation comparisons showed that moss cover significantly reduced soil pH at 3200, 3400, and 3500 m (*p* < 0.05), whereas no significant difference between the two treatments was observed at 3300 m (*p* > 0.05). Soil water content changed only slightly with increasing elevation in non-moss-covered soils and remained relatively stable overall. In moss-covered soils, soil water content decreased markedly from the highest level at 3200 m to 3300 m and then remained relatively stable from 3300 to 3500 m. Within each elevation, soil water content was significantly higher in moss-covered soils than in non-moss-covered soils (*p* < 0.05).

### 2.3. Moss Cover Alters SOC Fraction Allocation with Elevation-Dependent MNC Responses

**Figure 1:**
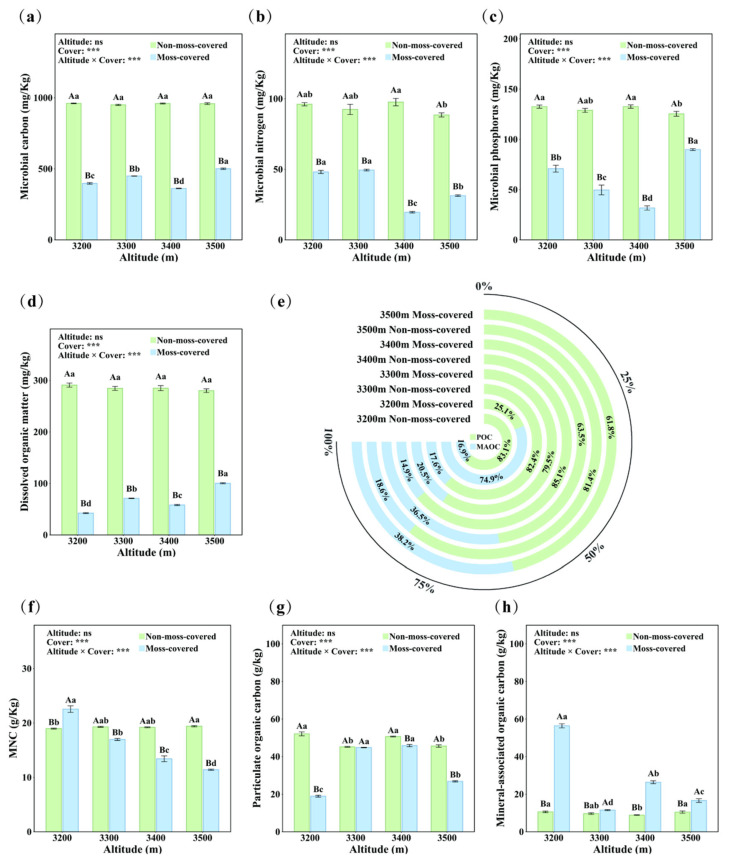
Effects of moss cover and elevation on (**a**) microbial biomass carbon, (**b**) microbial biomass nitrogen, (**c**) microbial biomass phosphorus, (**d**) dissolved organic matter, (**e**) the relative proportions of particulate organic carbon and mineral-associated organic carbon, (**f**) microbial necromass carbon, (**g**) particulate organic carbon, and (**h**) mineral-associated organic carbon. Bars represent means ± SE (*n* = 3). Different uppercase letters indicate significant differences between cover treatments within the same elevation, whereas different lowercase letters indicate significant differences among elevations within the same cover treatment (*p* < 0.05). The significance of the main effects of elevation and cover treatment and their interaction is shown in each panel. Here, ns indicates not significant; *** indicates significance at *p* < 0.001.

MB, DOM, MNC, and SOC fractions showed strong cover-dependent patterns that varied among elevations (Figure 1). Two-way ANOVA showed that the main effects of elevation on MBC, MBN, MBP, DOM, MNC, POC, and MAOC were not significant (*p* > 0.05), whereas both cover treatment and the elevation × cover treatment interaction had highly significant effects (*p* < 0.001). MBC changed only slightly with increasing elevation in non-moss-covered soils, whereas in moss-covered soils it showed a fluctuating pattern, increasing from 3200 to 3300 m, decreasing at 3400 m, and then increasing again at 3500 m. Within each elevation, MBC was significantly lower in moss-covered soils than in non-moss-covered soils (*p* < 0.05; Figure 1a). MBN changed only slightly with increasing elevation in non-moss-covered soils, with a slight decrease at 3500 m; in moss-covered soils, MBN remained relatively stable at first, then decreased markedly at 3400 m, and increased slightly at 3500 m. At each elevation, MBN was significantly lower in moss-covered soils than in non-moss-covered soils (*p* < 0.05; Figure 1b). MBP changed only slightly with increasing elevation in non-moss-covered soils, whereas in moss-covered soils it first decreased and then increased, declining from 3200 m to the lowest level at 3400 m and then increasing markedly at 3500 m. Within each elevation, MBP was significantly lower in moss-covered soils than in non-moss-covered soils (*p* < 0.05; Figure 1c). DOM remained relatively stable with increasing elevation in non-moss-covered soils, whereas in moss-covered soils it showed a fluctuating pattern, first increasing, then decreasing, and then increasing again, reaching the highest level at 3500 m. At each elevation, DOM was significantly lower in moss-covered soils than in non-moss-covered soils (*p* < 0.05; Figure 1d).

The proportions of SOC fractions also showed clear cover-dependent differences along the elevational gradient. In non-moss-covered soils, POC consistently dominated, while the proportion of MAOC remained relatively low and changed only slightly with increasing elevation. In moss-covered soils, the proportion of MAOC was markedly higher at 3200 m; thereafter, with increasing elevation, the proportion of MAOC decreased while that of POC increased correspondingly, and POC accounted for a relatively high proportion from 3300 to 3500 m (Figure 1e). MNC showed contrasting elevational patterns between the two cover treatments. It increased slightly with elevation in non-moss-covered soils but decreased continuously with elevation in moss-covered soils. Consequently, moss-covered soils had significantly higher MNC than non-moss-covered soils at 3200 m, but significantly lower MNC at 3300, 3400, and 3500 m (*p* < 0.05; Figure 1f). POC showed a fluctuating pattern with increasing elevation in non-moss-covered soils, with higher values at 3200 and 3400 m and lower values at 3300 and 3500 m. In moss-covered soils, POC first increased and then decreased with increasing elevation, rising from a lower level at 3200 m to higher levels at 3300–3400 m, and then decreasing at 3500 m. Within-elevation comparisons showed that, except at 3300 m where the difference between the two treatments was not significant (*p* > 0.05), POC was significantly lower in moss-covered soils than in non-moss-covered soils at the other elevations (*p* < 0.05; Figure 1g). MAOC changed only slightly with increasing elevation in non-moss-covered soils and remained at a relatively low level overall. In moss-covered soils, MAOC first decreased markedly with increasing elevation, then increased at 3400 m, and decreased again at 3500 m, with the highest value at 3200 m and the lowest at 3300 m. At each elevation, MAOC was significantly higher in moss-covered soils than in non-moss-covered soils (*p* < 0.05; Figure 1h). Overall, moss-covered soils generally showed lower POC and higher MAOC than non-moss-covered soils at most elevations.

### 2.4. Microbial Carbon-Cycling Functional Profiles Across Moss-Covered Elevational Groups and the Composite Non-Moss-Covered Reference Group

**Figure 2:**
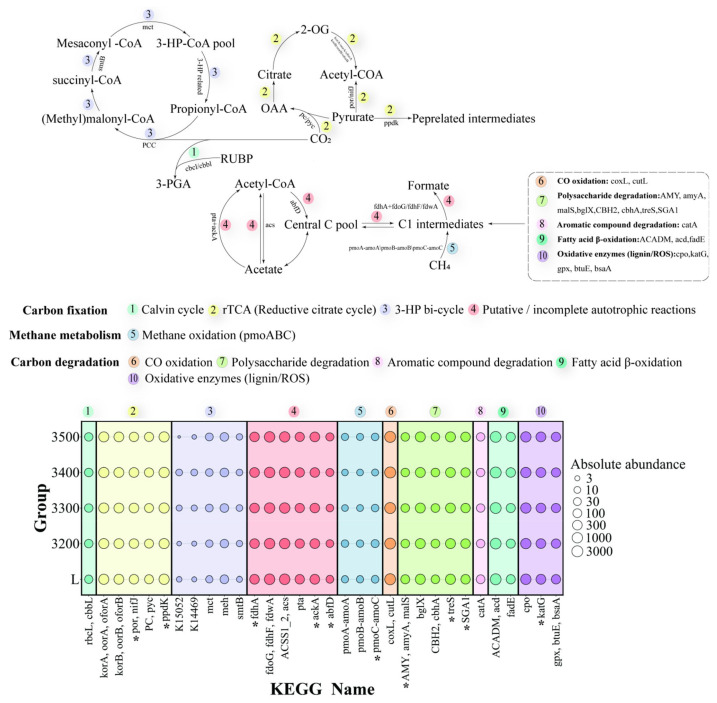
Conceptual map of soil carbon-cycling pathways and RPKM-normalized abundances of representative KEGG genes across moss-covered elevational groups and a composite non-moss-covered group (NMCG). Bubble size indicates gene abundance, and asterisks denote significant among-group differences based on uncorrected *p* values (*p* < 0.05).

Metagenomic analysis identified microbial carbon-cycling genes in all moss-covered elevational groups and in the composite non-moss-covered reference group, with the magnitude of among-group variation differing across functional modules (Figure 2). Because the non-moss-covered metagenomic samples were pooled across elevations to form the NMCG, comparisons between moss-covered groups and the NMCG should be interpreted as group-level functional differences rather than elevation-specific moss-cover effects. Genes associated with the Calvin cycle, rTCA cycle, 3-hydroxypropionate bi-cycle, CO oxidation, aromatic compound degradation, and fatty acid β-oxidation showed relatively limited variation among the moss-covered elevational groups and the composite non-moss-covered group (NMCG). In contrast, the genes showing more pronounced changes were mainly associated with C1 and acetate metabolism, methane oxidation, polysaccharide degradation, and oxidative enzyme-related processes. Among the C1 and acetate metabolism-related genes, *fdhA*/*fdoG*/*fdhF*/*fdwA*, *ackA*, and *abfD* generally maintained relatively high abundance in moss-covered soils; *ackA* and *abfD* slightly increased from 3200 m to 3300–3400 m and then decreased slightly at 3500 m. Compared with the NMCG, these genes showed similar or slightly higher relative abundances in the moss-covered groups, particularly at 3300–3400 m. Methane oxidation-related genes were generally less abundant, but *pmoC*/*amoC* first decreased from 3200 m to 3300–3400 m and then increased again at 3500 m. Compared with the NMCG, the relative abundance of *pmoC*/*amoC* was higher in the 3500 m moss-covered group. In the polysaccharide degradation module, *AMY*/*amyA*/*malS* remained highly abundant and changed only slightly with elevation, whereas *SGA1* generally increased with elevation and showed higher relative abundance at 3400–3500 m than in the NMCG. Among oxidative enzyme-related genes, katG generally increased with elevation in moss-covered soils and showed higher relative abundance at 3400–3500 m than in the NMCG. Overall, the differences between moss-covered groups and the NMCG were mainly observed in genes related to C1/acetate metabolism, polysaccharide degradation, methane oxidation, and oxidative enzyme-related processes.

**Figure 3:**
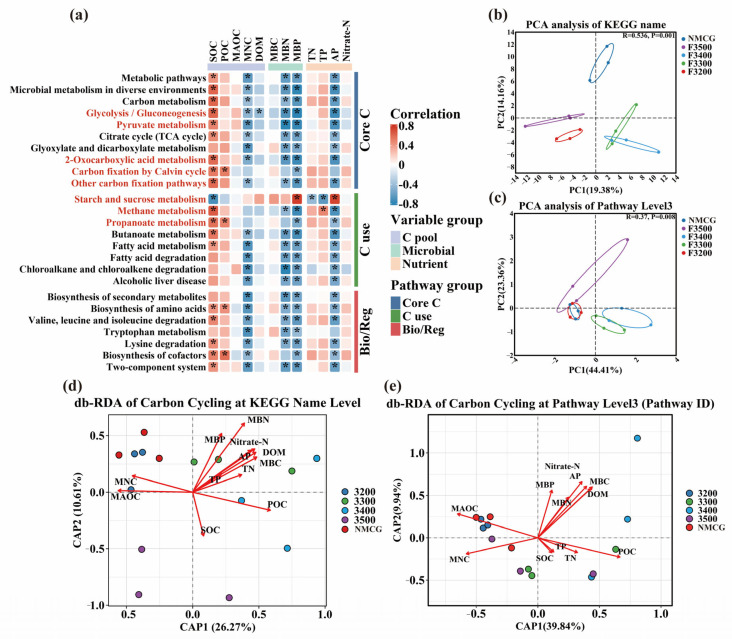
Correlation, PCA, and db-RDA analyses of soil carbon-related properties and microbial carbon-cycling functional profiles across moss-covered elevational groups (3200–3500 m) and the composite non-moss-covered reference group (NMCG). (**a**) Correlation analysis between soil carbon-related variables and microbial carbon-cycling functional profiles. (**b**) Principal component analysis (PCA) of microbial carbon-cycling functional profiles. (**c**) db-RDA ordination showing the relationships between soil carbon-related variables and microbial functional profiles. (**d**) db-RDA ordination showing the relationships between soil environmental variables and microbial functional profiles. Arrows indicate explanatory variables, and points represent individual samples or groups. The symbol * indicates significance at *p* < 0.05.

Carbon-cycling functional profiles showed clear covariation with soil carbon pools, nutrient availability, MB, and physicochemical properties (Figure 3). The correlation heatmap showed that different KEGG Level 3 carbon-cycling pathways had distinct association patterns with environmental factors and carbon-pool variables (Figure 3a). Among core carbon metabolism and carbon fixation pathways, most pathways, including metabolic pathways, microbial metabolism in diverse environments, carbon metabolism, glycolysis/gluconeogenesis, pyruvate metabolism, citrate cycle, 2-oxocarboxylic acid metabolism, carbon fixation by the Calvin cycle, and other carbon fixation pathways, were significantly positively correlated with SOC (*p* < 0.05). Among them, carbon fixation by the Calvin cycle was also significantly positively correlated with POC (*p* < 0.05). In contrast, these core carbon transformation pathways were mostly significantly negatively correlated with MNC, MBC, MBN, MBP, and AP (*p* < 0.05), and some pathways were also negatively correlated with DOM and nitrate-N. Higher relative abundances of core carbon metabolism and carbon fixation pathways were generally associated with higher SOC but lower MNC, MB, and available nutrient levels. Starch and sucrose metabolism was positively correlated with MBP and AP, but significantly negatively correlated with SOC, TN, and TP (*p* < 0.05). Propanoate metabolism was significantly positively correlated with SOC and POC (*p* < 0.05), but significantly negatively correlated with MBP and AP (*p* < 0.05). Butanoate metabolism, fatty acid metabolism, fatty acid degradation, chloroalkane and chloroalkene degradation, and alcoholic liver disease pathways were generally positively correlated with SOC, but were mostly significantly negatively correlated with MNC, MBN, MBP, and AP (*p* < 0.05). Among biosynthesis- and regulation-related pathways, biosynthesis of amino acids, valine, leucine and isoleucine degradation, lysine degradation, biosynthesis of cofactors, and two-component system were mostly significantly positively correlated with SOC or POC (*p* < 0.05), but significantly negatively correlated with MNC, MBN, MBP, and AP (*p* < 0.05). Overall, SOC was the main positive correlate of most core carbon metabolism, carbon fixation, and some biosynthesis pathways, whereas MNC, MB, and AP were the main negative correlates of these pathways.

The functional-gene-level heatmap showed broadly similar descriptive patterns to those observed at the pathway level (Figure S2). Because Figure S2 does not include significance markers, it mainly reflects the relative association strength between individual genes and environmental or carbon-pool variables. Overall, several carbon fixation and central carbon transformation genes, such as *coxL*/*cutL*, *fdoG*/*fdhF*/*fdwA*, *ACSS1_2*/*acs*, *por*/*nifJ*, *fdhA*, *rbcL*/*cbbL*, and *CBH2*/*cbhA*, showed stronger associations with SOC, MAOC, or MNC, but weaker associations with MBN, MBP, and AP, suggesting that these genes may be more closely related to stabilized carbon-pool formation and microbially processed carbon accumulation. In contrast, substrate-degradation-related genes, including *bglX*, *treS*, *AMY*/*amyA*/*malS*, and *catA*, showed stronger associations with DOM, MBC, MBN, MBP, and AP, but weaker associations with SOC, MAOC, and MNC, indicating that they may be more closely involved in labile organic matter decomposition, carbohydrate utilization, and available nutrient turnover. Methane oxidation-related genes, including *pmoA*/*amoA*, *pmoB*/*amoB*, and *pmoC*/*amoC*, were mainly more strongly associated with MBN, MBP, and AP, but showed weaker associations with SOC, suggesting that methane oxidation-related functions may be more closely related to microbial activity and nutrient availability. Overall, the gene-level correlation patterns were broadly consistent with the pathway-level analysis, although these descriptive associations should be interpreted cautiously because statistical significance was not indicated in Figure S2.

PCA ordination showed visual separation in carbon-cycling functional profiles between the moss-covered elevational groups and the NMCG (Figure 3b,c). At the KEGG name level, PC1 and PC2 explained 19.38% and 14.65% of the total variation, respectively (Figure 3b). The NMCG occupied a distinct region of the ordination space relative to most moss-covered groups, suggesting potential differences in carbon-cycling gene composition between the composite non-moss-covered reference group and moss-covered soils. However, because the NMCG represents pooled non-moss-covered samples across elevations, this separation should be interpreted as a difference between moss-covered elevational groups and the composite non-moss reference, rather than as evidence for elevation-specific moss-cover effects. Meanwhile, moss-covered groups at different elevations also showed some separation: the 3200 m and 3500 m groups were more inclined toward the negative PC1 direction, whereas the 3300 m and 3400 m groups were more inclined toward the positive PC1 direction, suggesting elevation-related differentiation in carbon-cycling gene composition under moss cover. At the Pathway Level 3, PC1 and PC2 explained 44.41% and 23.36% of the total variation, respectively, which was higher than that at the KEGG name level (Figure 3c). Group separation was clearer at the pathway level, particularly with the 3500 m group separating from the other groups along the positive PC2 direction, whereas the 3300 m and 3400 m groups were more associated with the positive PC1 direction. This descriptive pattern suggests that pathway-level profiles captured the among-group variation more clearly than individual gene-level profiles.

The db-RDA further showed that variation in carbon-cycling functional profiles was closely associated with soil carbon fractions, nutrient availability, and MB (Figure 3d,e). At the KEGG name level, CAP1 and CAP2 explained 26.27% and 10.61% of the variation, respectively (Figure 3d). POC, DOM, MBC, MBN, MBP, AP, nitrate-N, TN, and TP were mainly oriented toward the positive CAP1 direction, with MBN, MBP, AP, nitrate-N, DOM, and MBC showing similar directions. In contrast, MAOC and MNC were oriented toward the negative CAP1 direction and were opposite to POC and several labile nutrient and microbial biomass variables. SOC was mainly oriented toward the negative CAP2 direction and differed from some microbial biomass and available nutrient indicators. A similar pattern was observed at the Pathway Level 3, where CAP1 and CAP2 explained 39.84% and 9.94% of the variation, respectively (Figure 3e). POC, DOM, MBC, MBN, MBP, AP, and nitrate-N were mainly distributed along the positive CAP1 direction, whereas MAOC and MNC were located along the negative CAP1 direction, further indicating that particulate/labile carbon pools and mineral-associated carbon or MNC represented two contrasting gradients regulating carbon-cycling functions. Overall, variation in microbial carbon-cycling functional profiles among the MCGs and the NMCG was primarily associated with SOC fractions, nutrient availability, and MB. Functional profiles related to labile carbon use were more closely associated with DOM, POC, MB, and available nutrients, whereas another set of functional profiles was more closely aligned with MAOC and MNC. These ordination-based associations describe functional variation among moss-covered elevational groups and the composite NMCG, but they cannot resolve within-altitude functional variability in non-moss-covered soils.

### 2.5. Structural Equation Models Link Moss Cover and Elevation to Pathways Associated with Soil Carbon Stabilization

**Figure 4:**
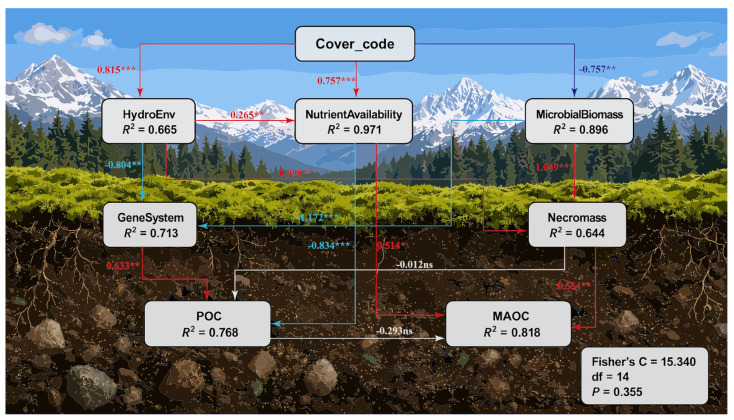
Hypothesis-guided piecewise structural equation model showing model-supported associations between moss cover and soil carbon transformation and stabilization. Cover_code denotes surface cover status; HydroEnv includes soil moisture, temperature and pH; nutrient availability includes TN, TP, AP, and NO_3^−^_-N; microbial biomass includes MBC, MBN, and MBP; GeneSystem represents carbon-cycling functional genes related to carbon fixation and degradation; microbial necromass is represented by MNC. Arrows indicate standardized path coefficients, with red and blue arrows showing positive and negative effects, respectively; dashed gray arrows indicate non-significant paths. Values in boxes indicate R^2^. Directed-separation test: Fisher’s C = 15.340, df = 14, *p* = 0.355, indicating no significant evidence of lack of fit under the specified exploratory path model. * *p* < 0.05, ** *p* < 0.01, *** *p* < 0.001; ns, not significant.

The structural equation model identified a network of model-supported associations linking moss cover with SOC allocation through the hydrothermal environment, nutrient availability, MB, MN, and the carbon-cycling gene system (Figure 4). The model showed an acceptable fit, with Fisher’s C = 15.340, df = 14, and *p* = 0.355. Cover_code had significant positive effects on HydroEnv (β = 0.815, *p* < 0.001) and the NutrientAvailability composite (β = 0.757, *p* < 0.001), but a significant negative effect on MicrobialBiomass (β = −0.757, *p* < 0.001). These paths showed that moss cover was associated with altered near-surface hydrothermal and nutrient-related conditions and with lower living MB. HydroEnv further had a significant positive effect on NutrientAvailability (β = 0.265, *p* < 0.01) and Necromass (β = 0.998, *p* < 0.01), but a significant negative effect on GeneSystem (β = −0.804, *p* < 0.001). MicrobialBiomass also showed contrasting effects on downstream microbial variables, with a significant negative effect on GeneSystem (β = −1.172, *p* < 0.001) and a significant positive effect on Necromass (β = 1.049, *p* < 0.001). These model-supported paths linked moss-associated variation in the soil microenvironment with nutrient-related conditions, MN accumulation, and the carbon-cycling functional gene system.

For downstream soil carbon pools, GeneSystem had a significant positive path to POC (β = 0.633, *p* < 0.01), indicating a positive model-supported association between microbial carbon-cycling functional potential and POC. Necromass was significantly positively associated with MAOC (β = 0.554, *p* < 0.01), whereas its direct effect on POC was not significant (β = −0.012, *p* > 0.05). This result suggests that MN contributed more strongly to MAOC formation than to POC accumulation, probably through mineral adsorption, aggregate protection, or organo-mineral association. However, the direct path from POC to MAOC was not significant (β = −0.293, *p* > 0.05), indicating that the model did not support a direct transformation from POC to MAOC. Overall, the model explained a large proportion of variance in HydroEnv (R^2^ = 0.665), NutrientAvailability (R^2^ = 0.971), MicrobialBiomass (R^2^ = 0.896), GeneSystem (R^2^ = 0.713), Necromass (R^2^ = 0.644), POC (R^2^ = 0.768), and MAOC (R^2^ = 0.818). Given the limited sample size, SEM pathways should be interpreted as model-supported associations rather than direct causal proof.

**Figure 5:**
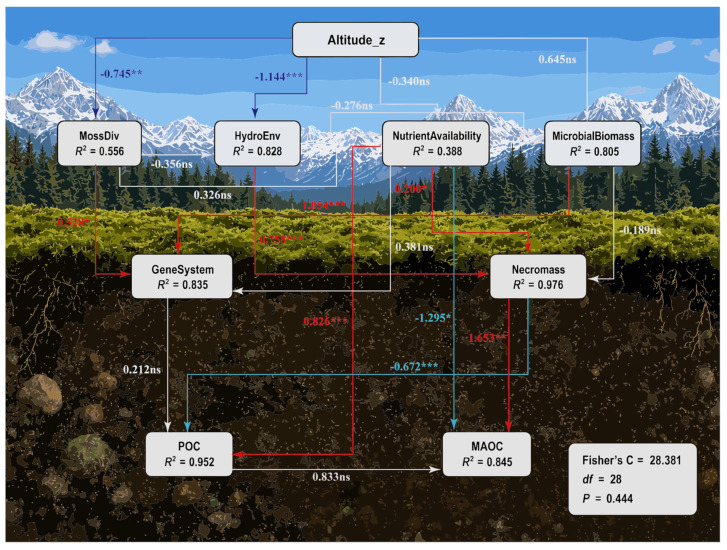
Hypothesis-guided piecewise structural equation model showing exploratory model-supported associations between elevation and soil carbon transformation and stabilization through moss diversity under moss-covered conditions. Altitude_z denotes standardized elevation; MossDiv represents moss diversity; HydroEnv includes soil moisture, temperature and pH; nutrient availability includes TN, TP, AP, and NO_3^−^_-N; microbial biomass includes MBC, MBN, and MBP; GeneSystem represents carbon-cycling functional genes related to carbon fixation and degradation; microbial necromass is represented by MNC. Arrows indicate standardized path coefficients, with red and blue arrows showing positive and negative effects, respectively, and gray arrows indicating non-significant paths. Values in boxes indicate R^2^. Directed-separation test: Fisher’s C = 28.381, df = 28, *p* = 0.444, indicating no significant evidence of lack of fit under the specified exploratory path model. * *p* < 0.05, ** *p* < 0.01, *** *p* < 0.001; ns, not significant.

Under moss-covered conditions, the hypothesis-guided piecewise SEM explored model-supported associations between elevation and soil carbon fractions through moss diversity, the hydrothermal environment, nutrient availability, MB, the carbon-cycling gene system, and MN formation (Figure 5). The directed-separation test did not indicate a significant lack of fit for the specified exploratory model (Fisher’s C = 28.381, df = 28, *p* = 0.444), but this result should be interpreted cautiously given the small sample size. Altitude_z had significant negative effects on MossDiv (β = −0.745, *p* < 0.01) and HydroEnv (β = −1.144, *p* < 0.001), indicating that increasing elevation was associated with reduced moss diversity and a marked shift in the near-surface hydrothermal environment. In contrast, the direct effects of Altitude_z on NutrientAvailability (β = −0.340, *p* > 0.05) and MicrobialBiomass (β = 0.645, *p* > 0.05) were not significant.

MossDiv had a significant positive effect on GeneSystem (β = 0.528, *p* < 0.05), suggesting that moss community diversity was closely linked to the carbon-cycling functional potential of soil microbial communities. MicrobialBiomass had a significant positive path to GeneSystem (β = 1.054, *p* < 0.001), indicating a positive model-supported association between living MB and the carbon-cycling functional gene system. However, the path from GeneSystem to POC was not significant (β = 0.212, *p* > 0.05), indicating that no significant direct association between these variables was supported by the model. HydroEnv had a significant positive path to Necromass (β = 0.758, *p* < 0.001), and NutrientAvailability also had a significant positive path to Necromass (β = 0.200, *p* < 0.05). In contrast, the path from MicrobialBiomass to Necromass was not significant (β = −0.189, *p* > 0.05). These paths indicated that MN accumulation was more strongly associated with hydrothermal and nutrient-related conditions than with current living MB in this model. In the downstream carbon-pool pathways, NutrientAvailability had a significant positive path to POC (β = 0.826, *p* < 0.001) but a significant negative path to MAOC (β = −1.295, *p* < 0.05), indicating contrasting model-supported associations with the two SOC fractions. By contrast, Necromass had a significant negative path to POC (β = −0.672, *p* < 0.001) and a significant positive path to MAOC (β = 1.653, *p* < 0.01). This pattern indicated a stronger positive association of MN with MAOC than with POC. The direct path from POC to MAOC was not significant (β = 0.833, *p* > 0.05), indicating that the model did not support a direct transformation from POC to MAOC.

Overall, several endogenous variables showed high R^2^ values in the specified model, including MossDiv (R^2^ = 0.556), HydroEnv (R^2^ = 0.828), NutrientAvailability (R^2^ = 0.388), MicrobialBiomass (R^2^ = 0.805), GeneSystem (R^2^ = 0.835), Necromass (R^2^ = 0.976), POC (R^2^ = 0.952), and MAOC (R^2^ = 0.845). These R^2^ values are reported descriptively and should not be interpreted as strong predictive validation because of the limited sample size.

## 3. Discussion

### 3.1. Altitudinal Variation in Moss-Layer Development and the Decoupling of Total and Available Nutrient Pools

Moss-layer growth traits, community α-diversity, and compositional similarity varied along the altitudinal gradient, but these responses did not follow a simple monotonic pattern. In this study, moss biomass increased with elevation and was significantly higher at 3400 and 3500 m than at 3200 and 3300 m (Figure S1a). In contrast, moss-layer thickness generally decreased with increasing elevation, although the pattern was not strictly monotonic. Specifically, moss-layer thickness was highest at 3200 m, decreased at 3300 m, slightly increased at 3400 m, and then reached the lowest value at 3500 m (Figure S1b). This indicates that greater moss biomass at higher elevations was not necessarily accompanied by a thicker moss layer, suggesting a partial decoupling between biomass accumulation and vertical layer development. Such a pattern may reflect changes in moss growth form, community composition, or layer compactness along the altitudinal gradient. Moss community α-diversity and compositional similarity also varied markedly among elevations. Chao1 richness and Shannon diversity were highest at 3200 m and declined toward 3500 m, where the lowest values were observed (Figure S1d,f). In contrast, the Simpson index increased toward the highest elevation and was significantly higher at 3500 m than at the low- and mid-elevation sites (Figure S1e). Dahl′s similarity index decreased markedly from 3200 m and remained lower at higher elevations, indicating reduced compositional similarity of moss communities along the altitudinal gradient (Figure S1c). These results suggest that altitude was associated with coordinated changes in moss biomass accumulation, layer structure, α-diversity, compositional similarity, and species turnover rather than with a uniform increase or decrease in moss growth. This interpretation is supported by evidence from forest bryophytes across 75 sites in the southeastern Qinghai–Tibet Plateau, where bryophyte diversity and distribution were strongly associated with climatic variables, especially minimum temperature and daily temperature range [42]. Their finding that temperature-related factors were dominant correlates of bryophyte diversity is consistent with our observation that moss diversity and layer traits changed nonlinearly along the altitudinal gradient.

Moss cover was associated with distinct changes in soil total nutrient pools, although SOC, TN, and TP did not respond synchronously along the altitudinal gradient. Compared with non-moss-covered soils, moss-covered soils had consistently higher SOC at all elevations (Table S3), indicating a positive association between moss cover and SOC storage. The largest difference in SOC occurred at 3200 m, where moss-layer thickness and soil water content were also highest (Figure S1b; Tables S3 and S4), suggesting that a thicker and wetter moss layer may contribute to SOC accumulation by improving water retention and buffering near-surface thermal conditions, although effects on organic matter turnover were not directly measured. This result is consistent with a global synthesis showing that soil mosses are associated with greater carbon sequestration and larger pools of key nutrients across broad environmental gradients [18], supporting a general association between moss cover and soil carbon and nutrient storage. More specifically, moss removal decreased surface SOC, TN, and soil water content, and moss traits together with soil water content regulated soil C and N accumulation [21]. This finding provides a plausible context for the relatively large SOC difference observed at 3200 m, although the contribution of moss-layer thickness and moisture cannot be isolated from other elevation-related factors. However, TN and TP did not increase uniformly under moss cover; instead, both showed elevation-dependent variation, reaching relatively high values at 3300 m and declining at 3500 m (Table S3). Therefore, moss cover did not simply increase all total nutrient pools simultaneously, but modified the accumulation patterns of different elemental pools according to altitude-associated environmental conditions.

Available nutrient concentrations showed more consistent differences between cover treatments than total nutrient pools and exhibited a pattern contrasting with that of SOC. Although moss-covered soils had higher SOC, they consistently showed lower nitrate-N and available phosphorus than non-moss-covered soils at all elevations (Table S3). This contrasting pattern indicates that higher SOC under moss cover was not accompanied by greater short-term nutrient availability. The structural equation models were consistent with this interpretation. In the overall model, surface-cover type had a significant path to NutrientAvailability, and this component showed a high R^2^ value (R^2^ = 0.971; Figure 4). Given the small sample size, this R^2^ value is reported descriptively rather than as strong predictive evidence. These model-supported paths suggest that variation in nitrate-N and available phosphorus was associated with both surface cover and the near-surface hydrothermal environment. In the moss-covered model, NutrientAvailability had a lower explained variance (R^2^ = 0.388), and the direct path from altitude to NutrientAvailability was not significant (Figure 5), indicating that altitude alone did not explain available nutrient variation within moss-covered soils. A previous study found that mosses reduced soil nitrogen availability in a subarctic birch forest through changes in the soil thermal regime and sequestration of deposited nitrogen, providing a possible explanation for the lower nitrate-N observed under moss cover in the present study [16]. For phosphorus, moss-growth areas had significantly lower pH and available phosphorus than non-moss-growth areas, and soil acidification and phosphorus deficiency were identified as major features of moss-growth soils; this is consistent with our observation of lower pH and AP under moss cover [43]. Moss mortality increased available P by 5–54% compared with living moss crusts [44], suggesting that biological uptake, immobilization, and pH- or enzyme-related processes may contribute to the lower AP under living moss cover; however, these processes were not directly measured in this study. Taken together, these results suggest that moss cover was associated with a redistribution of soil resources rather than a general increase in nutrient availability: moss-covered soils had higher SOC and altered total nutrient pools but lower nitrate-N and available phosphorus. These differences in nutrient availability and hydrothermal conditions may provide an environmental context for the contrasting responses of SOC fractions by influencing organic matter processing and microbial residue retention; however, their effects on turnover rates and mineral association require direct verification.

### 3.2. Moss Cover Alters SOC Fraction Allocation and Links MAOC Accumulation to Microbial Necromass

Moss cover reorganized SOC among active and mineral-associated pools rather than uniformly increasing soil carbon. Surface cover and its interaction with elevation significantly affected MBC, MBN, MBP, DOM, MNC, POC, and MAOC, whereas elevation alone had no consistent main effect (Figure 1). Across the elevational gradient, moss-covered soils contained less microbial biomass and DOM, generally less POC, but more MAOC than independently selected non-moss-covered soils. Thus, the higher SOC under moss cover was primarily associated with greater allocation to the mineral-associated fraction rather than enlargement of rapidly cycling carbon pools. This pattern is consistent with evidence that moss layers regulate soil C and N accumulation by modifying moisture, microbial habitats, and decomposition conditions [21], and that moss removal accelerates litter decomposition and nutrient release [45]. Nevertheless, these pool-size differences cannot independently distinguish changes in carbon inputs, decomposition, microbial use, or stabilization rates.

The hypothesis-guided structural equation models provided exploratory evidence that MAOC was more closely associated with microbial necromass than with the direct POC-to-MAOC path in the specified models. In the overall model, moss cover was significantly associated with HydroEnv, NutrientAvailability, and MicrobialBiomass, while HydroEnv and MicrobialBiomass were positively associated with Necromass. Necromass, in turn, had a significant positive path to MAOC (β = 0.554, *p* < 0.01; Figure 4). The lower microbial biomass observed under moss cover does not contradict this relationship because microbial biomass represents the living community at sampling, whereas MNC integrates microbial production, turnover, decomposition, and retention over time. Within moss-covered soils, HydroEnv and NutrientAvailability were positively associated with Necromass, which was positively associated with MAOC (β = 1.653, *p* < 0.01) but negatively associated with POC (Figure 5). In both models, the direct POC-to-MAOC pathway was not significant. These results are consistent with a closer association of MAOC with microbial residue retention than with direct transformation of the measured POC pool, although the models do not demonstrate physical transfer or causal mechanisms. [46,47]. This interpretation is consistent with studies identifying microbial assimilation, turnover, and necromass formation as important contributors to persistent SOC [48].

The effect of moss cover on MNC changed markedly with elevation because the two cover types followed contrasting elevational trajectories. Moss cover increased MNC at 3200 m, where the moss layer was thickest and soil water content, SOC, and the moss-induced differences in MNC and MAOC were greatest. Together with the positive HydroEnv-to-Necromass pathway, this pattern suggests that the moist and relatively buffered environment beneath the thick moss layer favored microbial residue accumulation and retention. Above 3200 m, however, moss-layer thickness and community diversity generally declined, and soil water content decreased sharply. Moss-covered soils also maintained lower microbial biomass, nitrate-N, and available phosphorus than non-moss-covered soils from the same elevational bands, potentially limiting microbial residue production and weakening the moss-associated retention advantage. The metagenomic profiles provide additional functional context: ackA and abfD were relatively abundant at 3300–3400 m, whereas SGA1 and katG increased toward higher elevations and pmoC/amoC rebounded at 3500 m. These shifts are consistent with enhanced acetate turnover, polysaccharide reprocessing, and oxidative transformation at middle and high elevations, which may promote microbial reuse of organic residues rather than their accumulation. Consequently, the balance under moss cover may have shifted from relatively strong residue retention at 3200 m toward lower residue production and/or greater residue reprocessing at 3300–3500 m. Because metagenomic abundance reflects functional potential rather than process rates, this explanation remains a data-supported interpretation rather than direct mechanistic evidence.

MNC and MAOC were therefore not fully synchronized along the elevational gradient. Although MNC declined continuously within moss-covered soils, MAOC remained higher under moss cover at every elevation and varied non-monotonically. This divergence suggests that microbial residue production and the subsequent retention of microbial-derived carbon in mineral-associated pools represent distinct processes. The POM-MAOM framework similarly emphasizes that particulate and mineral-associated organic matter differ in their formation and persistence [32]. Reactive Fe and Al phases may further enhance microbial residue retention and organo-mineral association [49,50]. But these mineral controls were not measured here. Overall, moss-covered soils were characterized by lower DOM, microbial biomass, and POC but higher MAOC, indicating contrasting carbon-pool patterns rather than direct carbon transfer from POC to MAOC. The strongest apparent microbial-residue retention occurred at 3200 m, where the moss layer was thicker and soil moisture was highest. The results are consistent with microbial necromass stabilization as a plausible link between moss-mediated environmental change and MAOC accumulation, rather than a demonstrated direct conversion of POC into MAOC.

### 3.3. Moss Cover Is Associated with a Decoupling Between Microbial Carbon Turnover and SOC Stabilization

The metagenomic results suggest that soil microorganisms had the functional potential to both produce microbial-derived carbon and reprocess organic matter. Carbon fixation and C1 metabolism genes, including *rbcL*/*cbbL*, *korA*, *oorA*/*oforA*, *por*/*nifJ*, *ppc*, *pyc*, *pmoA*/*pmoB*/*pmoC*, *coxL*/*cutL*, *fdh*/*fdo*, *acs*, and *pta*/*ackA*, indicate that microbial communities could incorporate CO_2_, CH_4_, CO, formate, acetate, and other small carbon compounds into central carbon metabolism (Figure 2). RuBisCO genes such as *rbcL*/*cbbL* are commonly interpreted as evidence of Calvin-cycle or RubisCO-related carbon fixation potential, whereas rTCA-related genes and carboxylases indicate the potential for autotrophic or mixotrophic carbon assimilation through acetyl-CoA, pyruvate, oxaloacetate, and citrate pools [51,52]. Similarly, pmoA is a common marker for aerobic methane-oxidizing microorganisms, and coxL is widely used to indicate microbial CO oxidation potential [53,54]. These genes indicate the genetic potential for microbial carbon assimilation and metabolism but do not directly quantify microbial growth, turnover, or necromass formation. However, the simultaneous presence of abundant organic matter degradation genes, including *AMY*/*amyA*/*malS*, *bglX*, *CBH2*/*cbhA*, *treS*/*SGA1*, *catA*, *ACADM*/*acd*/*fadE*, and *gpx*/*btuE*/*bsaA*/*katG*, indicates strong potential for polysaccharide degradation, aromatic compound degradation, fatty acid β-oxidation, and oxidative transformation (Figure 2). CAZyme-related genes are central to microbial decomposition of cellulose, hemicellulose, starch, and other plant-derived polymers, while aromatic degradation and oxidative stress-related enzymes facilitate the breakdown or transformation of more complex organic compounds [40,55,56]. Therefore, the simultaneous occurrence of assimilation- and degradation-related genes suggests the potential for both microbial carbon production and organic matter reprocessing. However, gene abundance alone cannot determine whether the associated processes occurred rapidly in situ.

The elevational pattern of carbon-cycling genes is consistent with the observed decoupling between MNC and MAOC. The strongest contrast occurred at 3200 m and 3500 m (Figure 1). At 3200 m, moss-covered soils showed relatively high MNC and MAOC but low POC, indicating that microbial necromass and mineral-associated carbon were simultaneously abundant at this elevation. In contrast, at 3500 m, non-moss-covered soils showed relatively high MBC, MNC, and POC but low MAOC. These contrasting standing stocks do not directly reveal whether microbial-derived carbon was retained, decomposed, or transferred among pools. The gene abundance patterns and ordination results should be interpreted as functional potential rather than direct evidence of carbon stabilization. Carbon fixation and C1 metabolism genes provide the potential source of microbial-derived carbon, whereas polysaccharide degradation, aromatic compound degradation, fatty acid β-oxidation, and oxidative enzyme genes indicate strong organic matter turnover potential (Figure 3 and Figure S2). Thus, the gene profiles provide a functional context for the coexistence of high MBC or MNC with low MAOC, but they do not identify the processes controlling microbial residue retention or MAOC formation. Previous studies have similarly emphasized that MN is an important source of SOC, but its persistence depends on mineral association, aggregate occlusion, and microbial re-utilization rather than on residue production alone [46,57,58,59]. Together, these studies are consistent with a mechanism in which MN accumulation depends not only on residue input, but also on decomposition loss and association with soil minerals.

The overall hypothesis-guided structural equation model further summarized model-supported associations among moss cover, the hydrothermal environment, nutrient availability, MB, GeneSystem, MN, and SOC fractions. In the full model, Cover_code was significantly associated with HydroEnv, NutrientAvailability, and MicrobialBiomass, and GeneSystem, POC, and MAOC showed relatively high R^2^ values of 0.713, 0.768, and 0.818, respectively (Figure 4). These values should be interpreted cautiously because the SEM was fitted as an exploratory model with a limited sample size. Importantly, GeneSystem had a significant positive path to POC, whereas Necromass had a significant positive path to MAOC (Figure 4). This result suggests that microbial functional potential was more closely associated with POC, whereas MAOC was more closely associated with MN in the model. The subsequent stabilization of MN was not directly measured. This is consistent with the MEMS framework, which proposes that stable SOM formation requires microbial processing of organic inputs followed by physicochemical protection, rather than direct preservation of plant residues alone [60]. Recent work also shows that MAOC accrual can be controlled more strongly by mineral preservation capacity than by microbial residue production alone [61,62]. Therefore, the full SEM was consistent with contrasting associations of GeneSystem and MN with POC and MAOC, respectively, but it did not demonstrate direct partitioning or transfer of microbial-derived carbon between these pools.

Under moss-covered conditions, the elevation-based SEM examined exploratory model-supported associations between altitude and SOC fractions through moss diversity, hydrothermal conditions, nutrient availability, GeneSystem, and MN-related pathways. Altitude_z significantly affected MossDiv and HydroEnv, while MossDiv was positively linked to GeneSystem, indicating that changes in moss communities along the elevational gradient could influence microbial carbon-cycling functional potential (Figure 5). The moss-covered model also showed high R^2^ values for GeneSystem, Necromass, POC, and MAOC, with values of 0.835, 0.976, 0.952, and 0.845, respectively (Figure 5). These high R^2^ values are reported descriptively and should not be interpreted as robust predictive validation under the small-sample design. Notably, NutrientAvailability had a significant positive path to POC and a significant negative path to MAOC, whereas Necromass had a significant positive path to MAOC (Figure 5). These paths indicate that NutrientAvailability was positively associated with POC, whereas MN was positively associated with MAOC within the moss-covered model. These contrasting associations provide a possible context for the coexistence of lower DOM and MB with higher MAOC under moss cover, although nutrient-release rates, microbial growth, and residue-preservation efficiency were not directly measured.

Several limitations should be considered when interpreting these findings. First, this observational study cannot establish causal relationships, and the SEM paths represent exploratory, model-supported associations. In addition, the relatively small sample size may reduce the power of directed-separation tests and can lead to apparently high R^2^ values or non-significant Fisher’s C results; therefore, the SEMs should not be interpreted as definitive validation of the proposed causal structure. Second, metagenomic gene abundance indicates functional potential rather than gene expression, enzyme activity, or actual carbon flux. Third, the NMCG comprised pooled non-moss-covered samples from different elevations; therefore, the metagenomic comparison cannot independently estimate elevation effects within non-moss-covered soils or provide a complete elevation × cover factorial analysis. Finally, reactive Fe and Al phases, aggregate structure, organo-mineral associations, microbial carbon-use efficiency, and isotope-based carbon transfer were not measured. Future studies combining these measurements with larger sample sizes will be necessary to verify the mechanisms underlying the observed MN-MAOC association.

Taken together, the metagenomic and SEM results support associations among moss cover, microbial carbon-cycling potential, MN, and SOC fraction allocation, rather than a simple relationship between larger microbial pools and greater MAOC. Carbon fixation and C1 metabolism genes suggest the potential formation of MB and microbial residues, whereas polysaccharide degradation, aromatic degradation, fatty acid β-oxidation, and oxidative enzyme genes help explain why high MBC and MNC may also reflect rapid carbon turnover rather than stable carbon accumulation (Figure 2 and Figure 3). The exploratory SEM results further showed model-supported positive paths from GeneSystem to POC and from Necromass to MAOC in the specified models (Figure 4 and Figure 5). Therefore, the coexistence of high MBC, MNC, and POC with low MAOC at some elevations may reflect a decoupling among microbial carbon production, turnover, and retention. In subalpine dark coniferous forests, moss cover was associated with lower POC, higher MAOC, and stronger model-supported links between MN and MAOC. These patterns may be related to moss-associated changes in microenvironmental and nutrient conditions, but they do not demonstrate a direct transfer of microbial-derived carbon from POC to MAOC.

## 4. Materials and Methods

### 4.1. Study Site and Experimental Design

**Figure 6:**
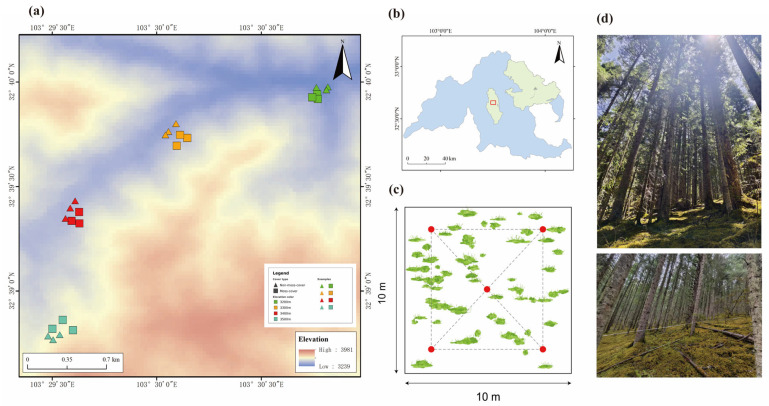
Study area, sampling-site distribution, field habitats, and plot-scale sampling design in the Erdaohai subalpine dark coniferous forest. (**a**) Spatial distribution of moss-covered and non-moss-covered sampling sites at 3200, 3300, 3400, and 3500 m a.s.l.; (**b**) geographical location of the Huanglong–Muni Valley study area in northwestern Sichuan, China; (**c**) schematic illustration of the 10 m × 10 m plot and five-point composite sampling design; (**d**) representative field photographs showing moss-covered and non-moss-covered microhabitats.

Field sampling was conducted in a representative subalpine dark coniferous forest in the Erdaohai Scenic Area, Songpan County, Sichuan Province, China (32°39′32.86″ N, 103°29′41.76″ E; Figure 6a,b). The region belongs to the cold climatic zone of the northwestern Sichuan Plateau, with a multi-year mean annual temperature of approximately 5.7 °C and mean annual precipitation of approximately 730 mm. To minimize systematic bias associated with topographic variation and stand heterogeneity, sampling sites were established along four elevational bands (3200, 3300, 3400, and 3500 m a.s.l.) within comparable mountain-slope positions. The plots were mainly located on north- to northeast-facing slopes, with slope angles ranging from 49°58′ to 58°58′. The sampled forest stands were dominated by *Picea* spp. and *Abies* spp., with approximately 80% spruce and 20% fir, and had comparable canopy closure ranging from 0.52 to 0.68. Sites with obvious recent disturbance were avoided during plot selection.

At each elevation, three independent 10 m × 10 m plots were established in moss-covered microhabitats. Within each plot, quadrats were arranged according to the layout shown in Figure 6c to record moss species composition and determine moss structural traits, including layer thickness and cover. Moss biomass was collected from representative quadrats, oven-dried to constant weight, and expressed as t ha^−1^ on a dry-weight basis. The overall sampling design, elevational distribution of sampling sites, field habitat characteristics, and quadrat arrangement at the plot scale are presented in Figure 6.

At each elevation, three independent moss-covered plots and three independent non-moss-covered plots were randomly selected within elevational bands where slope position, aspect, canopy closure, stand composition, and disturbance conditions were broadly comparable. The moss-covered and non-moss-covered plots were not arranged as one-to-one paired plots; instead, they represented independent plot-level replicates within each elevational band. Within each plot, five soil subsamples were collected from the 0–10 cm layer after carefully removing surface litter and the moss layer where present, and then homogenized to form one composite soil sample. Therefore, each composite soil sample, rather than each subsampling point, was treated as one independent field replicate. In total, 24 independent composite soil samples were collected for soil physicochemical properties and carbon-pool analyses, including three moss-covered and three non-moss-covered samples at each elevation. The same set of 24 samples was used for measurements of soil physicochemical properties, SOC fractions, microbial biomass carbon, microbial necromass carbon, POC, MAOC, and related carbon-pool indices, thereby ensuring that these variables shared the same replicate structure.

For the shotgun metagenomic analysis, moss-covered samples were analyzed by elevation, whereas non-moss-covered samples from the 3200–3500 m elevational range were consolidated into a composite non-moss-covered reference group, namely NMCG. This grouping was used to provide a common functional reference for non-moss-covered soils and to reduce the influence of small-scale stochastic variation on metagenomic signals. Consequently, three independent composite soil samples were prepared across this elevational range for sequencing and functional annotation. This methodological approach was based on the consideration that soil microbial communities and their functional potentials can be strongly influenced by spatial heterogeneity, which may obscure localized treatment effects [63,64,65,66]. In addition, the preparation of metagenomic samples and the selection of analytical workflows followed established guidelines for soil metagenomics and microbiome study design, including recommendations for quality control and potential pitfalls [67,68,69]. Therefore, the metagenomic data were analyzed among predefined groups and were not used to infer a full Elevation × Cover Type factorial response. All collected samples were placed in sterile containers, transported to the laboratory on dry ice, and stored at low temperatures until subsequent analysis.

### 4.2. Moss Sampling, Morphological Identification, and Community Diversity Analysis

Moss specimens were collected from each quadrat in the field and stored in individual paper packets labeled with the corresponding environmental metadata, including elevation, slope angle, microhabitat type, and moss cover. In the laboratory, moss specimens were identified based on morphological characteristics using stereoscopic and light microscopy. Identification focused on key diagnostic features, including leaf shape and apex, costa structure, laminal cell morphology, papillae, and, when present, sporophyte structures such as capsules and peristome teeth. Taxonomic identification was conducted with reference to regional floras, taxonomic keys, and standard bryophyte monographs. Voucher specimens were prepared and retained for future verification [70]. Taxonomic identification in this study was based exclusively on morphological characteristics.

Based on the identified moss species and their abundance in each quadrat, moss community α-diversity, compositional similarity, and pairwise β-diversity were calculated. The Shannon-Wiener and Gini-Simpson indices were used to characterize community diversity by incorporating species richness and evenness [71]. The Chao1 Estimator was applied to assess true species richness based on the proportion of rare taxa [72]. Dahl’s similarity index was also calculated to evaluate the compositional similarity of moss communities across sampling sites and elevational gradients [73]. In addition, pairwise β-diversity was assessed using the Cody and Wilson-Shmida indices to describe species turnover among moss communities at different elevations [74,75].

Differences in Shannon-Wiener, Gini-Simpson, and Chao1 indices among elevations were tested using one-way ANOVA, followed by Tukey’s HSD test for multiple comparisons when significant differences were detected. Dahl’s similarity index was used as a descriptive compositional-similarity measure, while Cody and Wilson-Shmida indices were calculated to describe pairwise species turnover among elevations.

### 4.3. Soil Physicochemical Properties and Carbon Fractionation

Soil pH, moisture, and temperature were determined as basic physicochemical indicators. Soil temperature was measured in situ at a depth of 5 cm using a digital probe thermometer (LCD-105, Yidu, Hengshui, China) before sample collection. Soil pH was determined using air-dried soil samples passed through a 2 mm sieve. Briefly, soil was mixed with deionized water at a soil-to-water ratio of 1:2.5 (*w*/*v*), shaken and equilibrated, and the pH of the suspension was measured using a calibrated pH meter (FE28-Standard, Mettler Toledo, Greifensee, Switzerland), following standard soil pH determination procedures [76]. Soil moisture content was determined gravimetrically using fresh field samples. Fresh soil was weighed immediately after collection, oven-dried at 105 °C to constant weight, and reweighed. Gravimetric soil moisture was calculated as the mass loss during drying divided by the oven-dry soil mass, following the conventional oven-drying method for soil water content determination [77,78].

Soil samples were air-dried at room temperature, and visible plant roots and stones were manually removed. The soil was then ground and passed through a 2 mm sieve. SOC and TN were determined by dry combustion using an elemental analyzer (UNICUBE, Elementar Analysensysteme GmbH, Langenselbold, Germany). For samples with high carbonate content, inorganic carbon was removed with dilute HCl prior to SOC analysis. TP was measured by the molybdenum blue colorimetric method after digestion with H_2_SO_4_ and HClO_4_ using a graphite digestion instrument (BYSM-24, Shanghai Bingyue Electronic Instrument Co., Ltd., Shanghai, China). AP was extracted with 0.5 M NaHCO_3_ (pH 8.5) and quantified colorimetrically using a visible spectrophotometer (UV752, Shanghai Yoke Instrument Co., Ltd., Shanghai, China). DOC and NO_3_^−^-N were extracted with deionized water and 2 M KCl, respectively, at a soil-to-solution ratio of 1:5 (*w*/*v*). After shaking and filtration through 0.45-μm membranes, DOC was analyzed using a total organic carbon analyzer (TOC-L, Shimadzu Corporation, Kyoto, Japan), and NO_3_^−^-N was analyzed using a continuous flow analyzer (AutoAnalyzer 3, Bran + Luebbe, Norderstedt, Germany).

Soil POC and MAOC were fractionated according to the wet-sieving method described by Cambardella and Elliott [79]. Briefly, soil samples were dispersed in 5 g L^−1^ sodium hexametaphosphate solution for 18 h and passed through a 53-μm sieve. The material retained on the sieve was defined as the POC fraction, whereas the fraction passing through the sieve was defined as the MAOC fraction. Both fractions were dried, ground, and analyzed for carbon content using an elemental analyzer (UNICUBE, Elementar Analysensysteme GmbH, Langenselbold, Germany).

MBC, MBN, and MBP were determined using the chloroform fumigation-extraction method [80,81]. MBC and MBN were extracted with 0.5 M K_2_SO_4_, while MBP was extracted with 0.5 M NaHCO_3_. Organic C in the K_2_SO_4_ extracts was measured using the same total organic carbon analyzer, and total N in the K_2_SO_4_ extracts was measured using the same continuous flow analyzer. MBP was quantified colorimetrically using the same visible spectrophotometer. The differences between fumigated and non-fumigated samples were converted to biomass pools using extraction efficiency factors of *k_EC_* = 0.45, *k_EN_* = 0.54, and *k_EP_* = 0.40.

MNC was estimated using amino sugars as biomarkers [82]. Soil samples were hydrolyzed with 6 M HCl at 105 °C for 8 h, purified, and derivatized to aldononitrile acetates. Glucosamine (GlcN) and muramic acid (MurA) were quantified using a gas chromatograph (Agilent 7820A, Agilent Technologies, Santa Clara, CA, USA). Fungal necromass C (FNC) and bacterial necromass C (BNC) were calculated based on the stoichiometric approaches proposed by Appuhn and Joergensen [83] and Liang [58], assuming a bacterial cell wall GlcN-to-MurA molar ratio of 2:1, and utilizing conversion factors of 45 for BNC and 9 for FNC.

### 4.4. Metagenomic Sequencing and Functional Annotation

Total genomic DNA was extracted from soil samples using the E.Z.N.A.^®^ Soil DNA Kit (Omega Bio-tek, Norcross, GA, USA) according to the manufacturer’s instructions. DNA integrity was evaluated using 1% agarose gel electrophoresis, and DNA concentration and purity were assessed using a NanoDrop 2000 spectrophotometer (Thermo Fisher Scientific, Waltham, MA, USA). Genomic DNA was fragmented to an average size of approximately 350 bp using a Covaris M220 focused ultrasonicator (Covaris, Woburn, MA, USA). Metagenomic libraries were constructed using the NEXTFLEX^®^ Rapid DNA-Seq Kit (Bioo Scientific, Austin, TX, USA) and sequenced on the DNBSEQ-T7 platform (MGI Tech Co., Ltd., Shenzhen, China) using a paired-end 150 bp strategy. Raw sequencing data were deposited in the NCBI Sequence Read Archive under BioProject accession number PRJNA1475836.

Raw reads were processed using fastp (v0.23.0) to remove adapter sequences and filter low-quality reads [84]. The documented workflow did not include a separate host-reference subtraction step. High-quality reads were de novo assembled into contigs using MEGAHIT (v1.2.9), retaining only contigs ≥ 500 bp [85]. Open reading frames (ORFs) were predicted from the assembled contigs using Prodigal (v2.6.3), and predicted genes with nucleotide lengths ≥ 100 bp were retained [86].

A non-redundant gene catalog was constructed using CD-HIT (v4.6.1), with the longest gene in each cluster retained as the representative sequence [87]. Quality-filtered reads from each sample were mapped back to this catalog using SOAPaligner (v2.21 release) [88], and gene abundance was quantified using the Reads Per Kilobase per Million mapped reads (RPKM) method. Representative sequences were aligned against the NCBI Non-redundant (NR) protein and KEGG databases (release 20241007) using DIAMOND (v2.0.13) [39,89]. KEGG Orthology annotations were used to identify genes associated with microbial carbon-cycling pathways.

Functional genes were classified according to their KEGG Orthology annotations. Genes were included in the carbon-cycling gene set when their KO annotations corresponded to the predefined functional categories examined in this study, including carbon fixation, C1 and acetate metabolism, methane oxidation, polysaccharide degradation, aromatic compound degradation, fatty acid β-oxidation, and oxidative transformation. For pathway-level analyses, RPKM-normalized gene abundances assigned to the same KEGG Pathway Level 3 category were aggregated within each sample to construct a pathway-level abundance matrix.

Because this shotgun metagenomic pipeline is based on DNA sequencing, the results reflect the genetic potential and functional composition of the microbial community rather than realized transcriptional activity, enzyme activity, or actual in situ metabolic fluxes. Therefore, metagenomic results were interpreted as microbial functional potential throughout this study.

Across the 15 shotgun metagenomic samples, raw reads ranged from 67.19 to 80.99 million, and 65.32–78.90 million clean reads were retained after quality filtering, corresponding to 9.78–11.81 Gb clean bases and base-retention rates of 95.19–97.21%. De novo assembly generated 254,383–474,606 contigs per sample, with total assembly lengths of 185.39–382.79 Mb and contig N50 values of 690–775 bp. Gene prediction identified 338,718–660,489 ORFs per sample, providing the basis for subsequent functional annotation and abundance analysis.

### 4.5. Statistical Analysis

All statistical analyses and data visualizations were performed in R version 4.5.1. Each composite soil sample was treated as an independent field replicate. For soil physicochemical properties and carbon-pool indices, including SOC, DOM, POC, MAOC, MNC, and MB, the effects of elevation, cover type, and their interaction were evaluated using two-way ANOVA based on the stratified random Elevation × Cover Type comparative framework. At each elevation, moss-covered and non-moss-covered plots were independently selected within elevational bands under comparable site conditions, rather than arranged as one-to-one paired plots. Therefore, no paired-block random effect was included in the statistical models. These analyses were based on 24 independent plot-level composite soil samples, including three moss-covered and three non-moss-covered samples at each elevation. Before analysis, normality and homogeneity of variance were checked. Where appropriate, simple-effect comparisons between moss-covered and non-moss-covered soils within each elevation were further conducted. Multiple comparisons were performed using Duncan’s multiple range test at *p* < 0.05.

For moss community data, differences in α-diversity indices among elevations were tested using one-way ANOVA, followed by Tukey’s HSD test for multiple comparisons when significant differences were detected. When the assumptions of normality or homogeneity of variance were not met, the Kruskal–Wallis test was used instead. Pairwise Cody β-diversity and Wilson-Shmida β-diversity indices were calculated to describe species turnover among moss communities at different elevations.

Functional dissimilarity between samples was calculated using Bray–Curtis distances based on RPKM-normalized abundance matrices. To assess the relationships between environmental variables and microbial functional profiles, distance-based redundancy analysis (db-RDA) with permutation tests [90] and canonical analysis of principal coordinates (CAP) [91] were performed. Spearman’s rank correlation analysis and ordinary least squares (OLS) regression were used to quantify associations among environmental factors, carbon-pool components, and key functional genes. Where multiple correlation tests were performed, *p*-values were adjusted using the Benjamini–Hochberg procedure where applicable. The concordance between microbial functional profiles and soil carbon-pool structure, such as POC-MAOC distribution, was further examined using Mantel tests and Procrustes analysis. Gene-level analyses were based on the RPKM abundance matrix of selected KO-annotated carbon-cycling genes, whereas pathway-level analyses were based on the corresponding KEGG Pathway Level 3 abundance matrix. PCA, Bray–Curtis dissimilarity, db-RDA, and correlation analyses were conducted separately for the two matrices.

Piecewise structural equation modeling (piecewise SEM) was used as an exploratory, hypothesis-guided analysis to evaluate whether the observed covariance structure was consistent with the proposed associations among surface cover, elevation, microenvironmental conditions, substrate availability, microbial functional potential, MN, and SOC fractions [92]. Piecewise SEM was conducted using the piecewiseSEM package version 2.3.1, and variance inflation factors (VIFs) were checked using the car package version 3.1.3 to assess collinearity among predictors. Because the GeneSystem variable was derived from the shotgun metagenomic dataset, SEM analyses were fitted only using samples with both soil-property and metagenomic information. The cover-associated SEM included 15 samples, consisting of 12 moss-covered samples from the four elevations and three composite non-moss-covered reference samples. Because the NMCG represented a composite non-moss-covered reference group, this model was used to evaluate cover-associated group-level pathways rather than a full Elevation × Cover Type factorial response. The moss-covered elevation SEM excluded the non-moss-covered reference group and included 12 moss-covered samples along the elevational gradient.

All continuous variables were standardized to z scores before model fitting. Composite variables were constructed by averaging z-scored indicators, and the direction of each composite variable was adjusted so that higher values consistently represented higher levels of the corresponding ecological process. HydroEnv was constructed from soil water content, soil temperature, and pH; NutrientAvailability was constructed from TN, TP, AP, and NO_3_^−^-N; MicrobialBiomass was constructed from MBC, MBN, and MBP; MossDiv was constructed from moss diversity indices; GeneSystem was constructed from RPKM-normalized carbon-cycling functional gene abundances; and microbial necromass was represented by MNC.

Model paths were specified a priori according to the conceptual framework that moss cover or elevation affects soil hydrothermal conditions, nutrient availability, microbial biomass, microbial functional potential, microbial necromass, POC, and MAOC. No automated stepwise path selection was performed, and nonsignificant paths were retained when they were part of the hypothesized pathway structure. Collinearity among predictors was checked for each component model, and variables with VIF values greater than 5 were not included in the same component model. For the cover-associated SEM, the component models were specified as follows: HydroEnv ~ Cover_code; NutrientAvailability ~ Cover_code + HydroEnv; MicrobialBiomass ~ Cover_code; GeneSystem ~ HydroEnv + MicrobialBiomass; Necromass ~ HydroEnv + MicrobialBiomass; POC ~ GeneSystem + Necromass; and MAOC ~ Necromass + POC. For the moss-covered elevation SEM, the component models were specified as follows: MossDiv ~ Altitude_z; HydroEnv ~ Altitude_z; NutrientAvailability ~ Altitude_z; MicrobialBiomass ~ Altitude_z; GeneSystem ~ MossDiv + MicrobialBiomass; Necromass ~ HydroEnv + NutrientAvailability + MicrobialBiomass; POC ~ GeneSystem + NutrientAvailability + Necromass; and MAOC ~ NutrientAvailability + Necromass + POC.

Directed-separation tests were performed using the basis set generated by piecewiseSEM, and model evaluation was based on Fisher’s C statistic, degrees of freedom, and the associated *p* value. Standardized path coefficients and R^2^ values of endogenous variables were reported. Given the observational design and limited sample size, piecewise SEM results were interpreted as exploratory model-supported associations rather than direct evidence of causality. Fisher’s C and R^2^ values were not used as evidence of excellent model fit; non-significant directed-separation tests were interpreted only as indicating no significant evidence of lack of fit under the specified model.

## 5. Conclusions

Across the 3200–3500 m elevational gradient, the moss layer shifted from a thick, moist, and diverse assemblage at lower elevations to a thinner, less diverse but more biomass-dominant layer at higher elevations. Moss biomass increased with elevation, whereas moss-layer thickness, species richness, and Shannon diversity generally declined. This reorganization coincided with changes in soil hydrothermal and nutrient conditions. Compared with independently selected non-moss-covered soils, moss-covered soils had lower mean soil temperature, higher soil water content and ΔT, and generally lower pH. Within moss-covered soils, soil water content and SOC were highest at 3200 m and generally declined with elevation. Moss cover increased SOC at every elevation but consistently reduced nitrate-N and available phosphorus, whereas TN and TP varied nonlinearly, reaching relatively high levels at 3300 m and declining markedly at 3500 m. Thus, the thicker and more diverse moss layer at lower elevation was associated with a wetter, carbon-rich but less nutrient-available near-surface environment that favored organic carbon retention, whereas moss-layer thinning, community simplification, and declining soil moisture at higher elevations altered substrate supply and the conditions for microbial carbon processing.

These surface changes did not uniformly enhance or suppress all microbial carbon-cycling functions; instead, they selectively reorganized specific functional modules. Core functions associated with carbon fixation and organic matter degradation remained broadly stable, whereas *ackA* and *abfD*, related to acetate and acetyl-CoA metabolism, were relatively abundant at 3300–3400 m. In contrast, the polysaccharide-degradation gene *SGA1* and the oxidative-transformation gene *katG* increased with elevation, while methane-oxidation-related *pmoC*/*amoC* rebounded at 3500 m. The metagenomic profile therefore shifted from greater potential for acetate turnover at mid-elevations toward greater potential for polysaccharide reprocessing and oxidative transformation at high elevation. This functional reorganization paralleled moss-layer thinning, community simplification, and changes in soil moisture and nutrient availability, providing a functional context for the decline in MNC with elevation under moss cover. By contrast, the thickest and wettest moss layer at 3200 m coincided with the highest MNC and MAOC, consistent with conditions more favorable for microbial necromass retention.

Taken together, the soil, moss, metagenomic, and exploratory structural equation modeling results suggest that the microbial functional gene system may represent an important link between variation in the moss layer and belowground carbon processing, but not the direct endpoint of MAOC accumulation. Moss diversity and microbial biomass were positively associated with GeneSystem, whereas MAOC was primarily associated with microbial necromass in both models, and the direct POC-to-MAOC pathway was not significant. Stable-carbon accumulation under moss cover was therefore not explained simply by greater microbial biomass or a higher abundance of carbon-cycling genes. Rather, moss cover altered the fate of microbially processed carbon across production, decomposition, reuse, and retention: active carbon pools and nutrient availability declined, whereas retained microbial residues were more strongly associated with the mineral-bound carbon pool. Overall, moss-covered soils were characterized by lower DOM, microbial biomass, and generally POC but higher MAOC, indicating contrasting carbon-pool patterns rather than direct carbon transfer from POC to MAOC.

## Data Availability

The data used and presented in this study are included in the main text and Supplementary Materials. The raw sequencing data have been submitted to NCBI under submission number PRJNA1475836.

## Supplementary Materials

The following supporting information can be downloaded at: https://www.mdpi.com/article/10.3390/plants15132098/s1, Figure S1: Variations in moss layer characteristics and α-diversity indices under spruce forests across different altitudes. (A) Moss layer biomass (t hm^−2^); (B) Moss layer thickness (cm); (C) Dahl index; (D) Chao1 index; (E) Simpson index (1-D); (F) Shannon index. In bar charts (A,B), data are presented as mean ± standard error (SE), and different lowercase letters indicate significant differences among altitudes (Duncan’s multiple range test, *p* < 0.05). In boxplots (C–F), the box represents the interquartile range (IQR), the horizontal line within the box indicates the median, and the whiskers represent the data range. Asterisks indicate significant differences between altitudes (*, *p* < 0.05; **, *p* < 0.01; ***, *p* < 0.001); Figure S2: Heatmap showing the association patterns between carbon-cycling functional genes and soil environmental, nutrient, microbial biomass, and carbon-pool variables. Genes were grouped into carbon fixation, carbon metabolism, and methane metabolism modules according to their annotated functions. Colors indicate Z-score-standardized values, with red representing higher relative association intensity and blue representing lower relative association intensity. DOM, dissolved organic matter; MBC, microbial biomass carbon; MBN, microbial biomass nitrogen; MBP, microbial biomass phosphorus; AP, available phosphorus; TN, total nitrogen; TP, total phosphorus; POC, particulate organic carbon; Nitrate-N, nitrate nitrogen; SOC, soil organic carbon; MAOC, mineral-associated organic carbon; MNC, microbial necromass carbon; Table S1: Pairwise Cody β-diversity index (βC) of moss communities among different elevations; Table S2: Pairwise Wilson–Shmida β-diversity index (βT) of moss communities among different elevations; Table S3: Soil physicochemical properties under different moss cover treatments along the elevational gradient; Table S4: Variations in soil microclimatic and physicochemical properties under moss-covered and non-moss-covered soils across different elevations.

## Abbreviations

The following abbreviations are used in this manuscript:

SOC	Soil organic carbon
SOM	Soil organic matter
TN	Total nitrogen
TP	Total phosphorus
AP	Available phosphorus
NO_3_^−^-N	Nitrate nitrogen
DOM	Dissolved organic matter
DOC	Dissolved organic carbon
POC	Particulate organic carbon
MAOC	Mineral-associated organic carbon
POM	Particulate organic matter
MAOM	Mineral-associated organic matter
MB	Microbial biomass
MBC	Microbial biomass carbon
MBN	Microbial biomass nitrogen
MBP	Microbial biomass phosphorus
MN	Microbial necromass
MNC	Microbial necromass carbon
FNC	Fungal necromass carbon
BNC	Bacterial necromass carbon
GlcN	Glucosamine
MurA	Muramic acid
MCG	Moss-covered group
NMCG	Non-moss-covered composite group
KEGG	Kyoto Encyclopedia of Genes and Genomes
RPKM	Reads per kilobase per million mapped reads
NCBI	National Center for Biotechnology Information
SRA	Sequence Read Archive
CAZyme	Carbohydrate-active enzyme
PCA	Principal component analysis
db-RDA	Distance-based redundancy analysis
CAP	Canonical analysis of principal coordinates
SEM	Structural equation modeling
ANOVA	Analysis of variance
SE	Standard error
MEMS	Microbial Efficiency-Matrix Stabilization
