# Supplementary material for: Moss Cover Redirects Soil Organic Carbon from Active Turnover to Mineral-Associated Stabilization in Subalpine Forests
Source: Plants (Basel). 2026 Jul 6;15(13):2098. doi: 10.3390/plants15132098 (PMC13363821; doi:10.3390/plants15132098)
Supplement: Supplementary file 1 [file plants-15-02098-s001.zip › plants-4379331-supplementary.pdf]

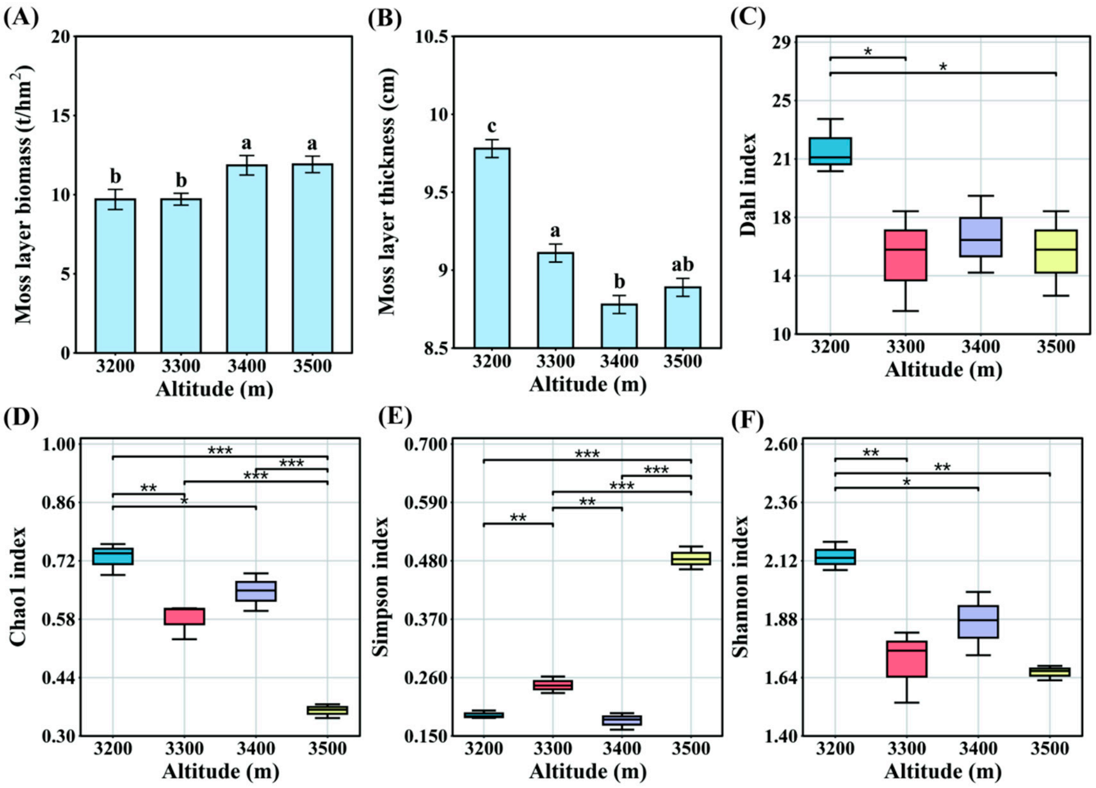

Figure S1. Variations in moss layer characteristics and α-diversity indices under spruce forests across different altitudes. (A) Moss layer biomass (t hm^{-2}); (B) Moss layer thickness (cm); (C) Dahl index; (D) Chao1 index; (E) Simpson index (1-D); (F) Shannon index. In bar charts (A, B), data are presented as mean ± standard error (SE), and different lowercase letters indicate significant differences among altitudes (Duncan's multiple range test, $P < 0.05$). In boxplots (C–F), the box represents the interquartile range (IQR), the horizontal line within the box indicates the median, and the whiskers represent the data range. Asterisks indicate significant differences between altitudes (*, $P < 0.05$; **, $P < 0.01$; ***, $P < 0.001$).

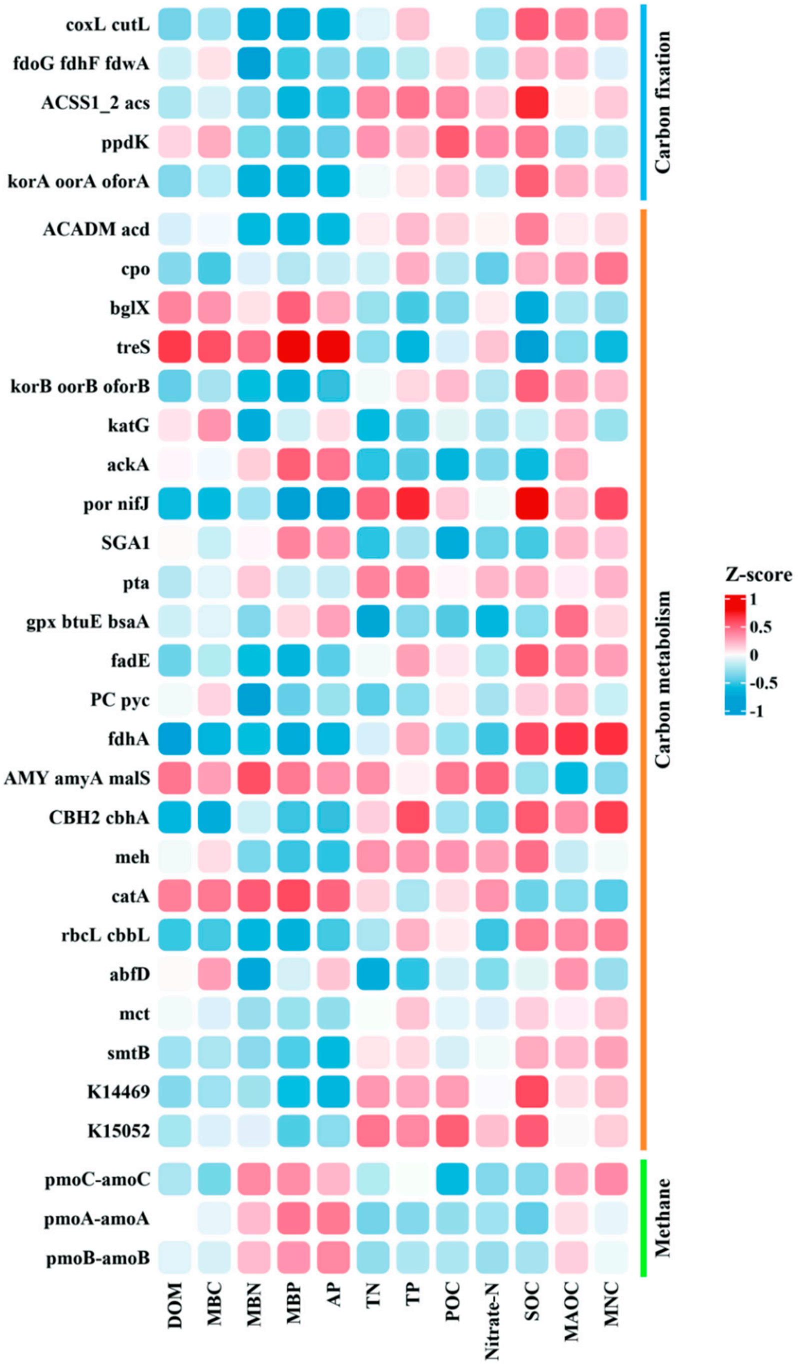

Figure S2. Heatmap showing the association patterns between carbon-cycling functional genes and soil environmental, nutrient, microbial biomass, and carbon-pool variables.Genes were grouped into carbon fixation, carbon metabolism, and methane metabolism modules according to their annotated functions. Colors indicate Z-score-standardized values, with red representing higher relative association intensity and blue representing lower relative association intensity. DOM, dissolved organic matter; MBC, microbial biomass carbon; MBN, microbial biomass nitrogen; MBP, microbial biomass phosphorus; AP, available phosphorus; TN, total nitrogen; TP, total phosphorus; POC, particulate organic carbon; Nitrate-N, nitrate nitrogen; SOC, soil organic carbon; MAOC, mineral-associated organic carbon; MNC, microbial necromass carbon.

Table S1. Pairwise Cody β-diversity index (βC) of moss communities among different elevations.

Elevation (m)	3200	3300	3400	3500
3200	-	8.00	10.50	9.51
3300	8.00	-	9.50	9.52
3400	10.50	9.50	-	7.11
3500	9.51	9.52	7.11	-

* Values represent pairwise Cody β-diversity indices of moss communities among four elevations (3200, 3300, 3400, and 3500 m a.s.l.). Higher βC values indicate greater species turnover and larger differences in community composition between elevations. The diagonal "–" indicates comparisons within the same elevation and was not calculated.

Table S2. Pairwise Wilson–Shmida β-diversity index (βT) of moss communities among different elevations.

Elevation (m)	3200	3300	3400	3500
3200	-	0.64	1.02	1.03
3300	0.64	-	0.93	1.02
3400	1.02	0.93	-	1.00
3500	1.03	1.02	1.00	-

* Values represent pairwise Wilson–Shmida β-diversity indices of moss communities among four elevations (3200, 3300, 3400, and 3500 m a.s.l.). Higher βT values indicate greater species turnover and more pronounced differences in moss community composition between elevations. The diagonal "–" indicates comparisons within the same elevation and was not calculated.

Table S3 Soil physicochemical properties under different moss cover treatments along the elevational gradient.

Elevation (m)	Treatment	SOC (g kg^{-1})	TN (g kg^{-1})	TP (g kg^{-1})	Nitrate-N (mg kg^{-1})	AP (g kg^{-1})	DOM (mg kg^{-1})
3200	non-moss-cover	63.53 ± 2.02 ^{Ac}	8.598 ± 0.294 ^{Aa}	0.962 ± 0.007 ^{Ac}	3.25 ± 0.10 ^{Ac}	0.347 ± 0.003 ^{Aa}	290.96 ± 7.00 ^{Aa}
	Moss-covered	136.35 ± 5.53 ^{Bb}	6.626 ± 0.537 ^{Bb}	1.017 ± 0.065 ^{Ab}	0.28 ± 0.03 ^{Bc}	0.080 ± 0.002 ^{Bab}	42.39 ± 1.44 ^{Bd}
3300	non-moss-cover	56.34 ± 4.51 ^{Aa}	8.282 ± 0.176 ^{Aa}	1.076 ± 0.038 ^{Ab}	3.83 ± 0.08 ^{Aa}	0.343 ± 0.004 ^{Aa}	284.66 ± 6.73 ^{Aa}
	Moss-covered	99.21 ± 3.82 ^{Ba}	9.084 ± 0.866 ^{Aa}	1.186 ± 0.091 ^{Aa}	0.81 ± 0.05 ^{Ba}	0.090 ± 0.001 ^{Bc}	71.12 ± 0.99 ^{Bb}
3400	non-moss-cover	62.01 ± 5.02 ^{Ab}	7.302 ± 0.138 ^{Ab}	1.200 ± 0.034 ^{Aa}	3.58 ± 0.21 ^{Ab}	0.374 ± 0.010 ^{Ab}	285.05 ± 8.40 ^{Aa}
	Moss-covered	99.92 ± 6.56 ^{Ba}	7.305 ± 0.645 ^{Ab}	0.994 ± 0.033 ^{Bb}	0.46 ± 0.03 ^{Bb}	0.142 ± 0.003 ^{Bb}	58.13 ± 1.87 ^{Bc}
3500	non-moss-cover	60.87 ± 3.63 ^{Ab}	7.768 ± 0.311 ^{Ab}	0.935 ± 0.009 ^{Ac}	3.96 ± 0.10 ^{Aa}	0.377 ± 0.007 ^{Aa}	280.13 ± 6.10 ^{Aa}
	Moss-covered	86.54 ± 5.43 ^{Bd}	4.835 ± 0.466 ^{Bc}	0.707 ± 0.034 ^{Bc}	0.46 ± 0.01 ^{Bb}	0.133 ± 0.003 ^{Ba}	100.42 ± 1.80 ^{Ba}

* Values are presented as mean ± standard error (n = 3).Abbreviations: SOC, soil organic carbon; TN, total nitrogen; TP, total phosphorus; AP, available phosphorus; DOM, dissolved organic matter. Different uppercase letters (A, B) indicate significant differences between treatments (non-moss-covered soil vs. Moss-covered) at the same altitude (P < 0.05). Different lowercase letters (a–d) indicate significant differences among different altitudes within the same treatment (P < 0.05).

Table S4. Variations in soil microclimatic and physicochemical properties under moss-covered and non-moss-covered soils across different elevations.

Elevation (m)	Treatment	Mean temperature (°C)	ΔT (°C)	pH	Soil water content (%)
3200	non-moss-cover	10.20 ± 0.26^{Ac}	4.50 ± 0.10^{Ba}	5.76 ± 0.07^{Aa}	23.14 ± 1.41^{Ba}
	Moss-cover	9.46 ± 0.18^{Ba}	6.71 ± 0.23^{Aa}	4.67 ± 0.23^{Bc}	50.80 ± 4.97^{Aa}
3300	non-moss-cover	10.43 ± 0.15^{Ac}	4.33 ± 0.06^{Ba}	5.84 ± 0.05^{Aa}	20.85 ± 1.56^{Ba}
	Moss-cover	9.11 ± 0.10^{Bab}	6.26 ± 0.21^{Aab}	5.74 ± 0.13^{Aa}	34.38 ± 3.46^{Ab}
3400	non-moss-cover	11.27 ± 0.06^{Ab}	3.77 ± 0.15^{Bb}	5.74 ± 0.05^{Aa}	23.57 ± 2.00^{Ba}
	Moss-cover	8.24 ± 0.09^{Bc}	6.13 ± 0.15^{Ab}	4.55 ± 0.16^{Bc}	35.37 ± 2.73^{Ab}
3500	non-moss-cover	11.90 ± 0.10^{Aa}	3.00 ± 0.26^{Bc}	5.84 ± 0.09^{Aa}	24.25 ± 1.37^{Ba}
	Moss-cover	8.98 ± 0.13^{Bb}	4.42 ± 0.28^{Ac}	5.18 ± 0.19^{Bb}	35.71 ± 2.07^{Ab}

* Values are presented as mean ± standard deviation. Different uppercase letters indicate significant differences between moss-covered and non-moss-covered soils at the same elevation, whereas different lowercase letters indicate significant differences among elevations within the same treatment for the same variable ($P < 0.05$). ΔT represents soil temperature variation.
